# Burnout and Emotions at Work: The Moderating Role of Guilt in a Cross-National Sample

**DOI:** 10.3390/ejihpe16100157

**Published:** 2026-10-07

**Authors:** Jonathan Peñalver, Hugo Figueiredo-Ferraz, Wenceslao Unanue, Deissy Johana Correa Estupiñán, María del Pilar Gómez Carreño

**Affiliations:** 1 WANT Research Team, Universitat Jaume I, 12071 Castellón de la Plana, Spain; jpenalve@uji.es; 2 Facultad de Ciencias Sociales y Jurídicas, Universidad Internacional de Valencia (VIU), 46002 Valencia, Spain; 3 Business School, Universidad Adolfo Ibáñez, Av. Diag. Las Torres 2640, Peñalolén 7941169, Santiago, Chile; wenceslao.unanue@uai.cl; 4 Corporación Universitaria Iberoamericana (IBERO), Cl. 67 #5-27 Sede Principal, Bogotá 110231, Colombia; deissy.correa@docente.ibero.edu.co; 5 Campus Ciudad de México, Business School, Tecnológico de Monterrey, Monterrey 14380, Mexico; pilar.gomcar@tec.mx

**Keywords:** burnout, emotions at work, guilt, moderation, cross-national

## Abstract

Burnout is a prevalent occupational phenomenon with significant consequences for employee well-being. Although emotions have been studied in relation to burnout, less attention has been paid to their function as burnout outcomes and to the role of guilt as a boundary condition of this process. This study examined the relationships between burnout and positive and negative emotions at work, as well as the moderating role of guilt, from a cross-national and gender perspective. A sample of 857 employees (57.2% women; M age = 38.50 years) from Spain, Chile, Colombia, and Mexico completed validated measures of burnout (Spanish Burnout Inventory), emotions at work, and guilt. Structural equation modelling and multigroup analyses were conducted, complemented by moderation analyses using the PROCESS macro. Burnout was negatively associated with positive emotions and positively associated with negative emotions. Multigroup analyses supported strict invariance across gender and configural–metric invariance across countries. Guilt significantly moderated both relationships, such that the associations between burnout and emotional experiences were weaker at higher levels of guilt. At comparable levels of burnout, higher guilt was associated with less favorable predicted emotional levels, indicating that guilt shapes the strength, rather than the direction, of the burnout–emotion association. These findings position guilt as a boundary condition of the burnout–emotion association, with implications for burnout prevention and employee well-being interventions.

## 1. Introduction

Employee well-being has become a central topic of interest for both researchers and practitioners due to its wide-ranging consequences for individuals, organizations, and society. For instance, recent reports indicate that global employee engagement fell to 21% in 2024, with the associated loss of productivity costing the world economy approximately $438 billion (Gallup, 2025). Furthermore, according to the World Health Organization, depression and anxiety lead to an estimated 12 billion lost working days each year, corresponding to approximately US$1 trillion in lost productivity worldwide (World Health Organization, 2024). In this context, the literature is clear: unfavorable working conditions reduce positive work experiences and increase the likelihood of negative psychological states in the workplace, among which burnout has received considerable scholarly attention (Bakker et al., 2023).

Burnout has been defined in multiple ways. Maslach et al. (2001) defined burnout as a prolonged response to chronic emotional and interpersonal stressors on the job, characterized by three dimensions: exhaustion, cynicism, and inefficacy. Similarly, Bakker et al. (2014) described burnout as a state of exhaustion and cynicism toward work. Later, Gil-Monte (2011) conceptualized burnout as a syndrome resulting from chronic occupational stress of an interpersonal nature. Despite these different definitions, one fact is clear: in 2019, burnout syndrome was formally incorporated into the 11th Revision of the International Classification of Diseases (ICD-11) as an occupational phenomenon (World Health Organization, 2019). Both before and after its formal recognition in the ICD-11, burnout has generated substantial concern among researchers and practitioners, resulting in a growing body of studies examining its prevalence across a wide range of occupational groups. For example, evidence from systematic reviews and meta-analyses has revealed particularly high prevalence rates among professionals such as teachers, social workers, and nurses (García-Carmona et al., 2019; Giménez-Bertomeu et al., 2024; Woo et al., 2020). In other words, burnout should be acknowledged as a major public health issue (Spencer-Hwang et al., 2024).

According to the burnout model proposed by Gil-Monte (2005), employees gradually lose enthusiasm toward their work, experience psychological exhaustion, and develop indifferent attitudes toward the people with whom they interact. For some individuals, this process is further accompanied by feelings of guilt arising from the recognition that their attitudes and behaviors conflict with their professional values, resulting in a more severe burnout profile. Burnout has been associated with a wide range of negative consequences across different domains, including cognitive (e.g., diminished self-esteem and feelings of helplessness), attitudinal (e.g., indifference and cynicism), behavioral (e.g., social withdrawal and aggression), physiological (e.g., sleep disturbances, fatigue, and hypertension), and emotional (e.g., irritability and aversion).

### 1.1. Burnout and Emotions at Work

Emotions, defined as brief, specific, and intense affective reactions in the workplace (Barsade & Gibson, 2007), play a key role in a broad range of biological, social, cognitive, and health-related processes (Barrett et al., 2016). Specifically, the literature has shown that various emotion-related constructs, such as emotion regulation, emotional labor, and emotional intelligence, are significantly associated with burnout (Braun et al., 2020; Kariou et al., 2021; Soriano-Sánchez & Jiménez-Vázquez, 2023). Consistent with these findings, early research demonstrated that positive and negative emotions are directly and significantly related to the development of burnout (Zapf et al., 2001). Subsequent studies have reinforced this perspective across different occupational groups, including teachers (Atmaca et al., 2020) and nurses (Szczygieł & Mikołajczak, 2018). For instance, Atmaca et al. (2020), in a study involving 564 in-service teachers, found that positive and negative emotions at work significantly predicted educators’ levels of burnout. Specifically, love was negatively associated with burnout, suggesting that experiencing this emotion may act as a protective factor against exhaustion. In contrast, sadness and fear showed positive associations, indicating that these emotions increase the risk of burnout.

However, a smaller body of research suggests that the causal direction may operate in the opposite way. For instance, using a two-wave panel design with 941 school teachers, Burić et al. (2019) examined the reciprocal relationships between burnout, negative emotions, and psychopathological symptoms over a six-month period. Contrary to expectations, the results did not show that negative emotions predicted burnout over time. Instead, burnout emerged as a significant predictor of negative emotions, indicating that teachers experiencing higher levels of burnout subsequently reported more anger, frustration, hostility, and hopelessness toward their students. These findings are also consistent with Gil-Monte’s (2005) theoretical model of burnout, which conceptualizes emotional reactions (e.g., irritability and aversion) as outcomes of a progressive process of psychological deterioration. Therefore, we hypothesize that

**Hypothesis** **1** **(H1).** *Burnout will be negatively associated with positive emotions at work.*

**Hypothesis** **2** **(H2).** *Burnout will be positively associated with negative emotions at work.*

To date, empirical evidence has documented gender and cross-national differences in burnout levels. Regarding gender, research has produced mixed findings. While some studies have reported higher levels of emotional exhaustion among women and higher levels of depersonalization among men, the magnitude of these differences is generally small and inconsistent across occupational groups and cultural contexts (Purvanova & Muros, 2010). Moreover, evidence suggests that gender differences are more likely to reflect variations in symptom intensity than differences in the underlying nature of burnout itself. Supporting this view, psychometric studies have demonstrated measurement invariance of burnout instruments across gender, indicating that burnout is conceptualized and assessed similarly in men and women (Osca et al., 2024; Umaña-Ruiz et al., 2023).

Regarding country differences, an international study of nursing staff found that exhaustion was higher in Japan, whereas cynicism and intentions to leave the profession were greater in Malaysia. In contrast, Thailand showed lower levels of burnout and turnover intentions, together with higher professional efficacy, compared to the United States and Canada (Ohue et al., 2021). However, a meta-analytic review of 58 samples from eight Latin American countries suggested that these differences may be explained by both occupation and language (García-Arroyo & Osca Segovia, 2018). Furthermore, Gil-Monte et al. (2023) supported the cross-cultural validity and measurement invariance of burnout measures, suggesting that the fundamental dimensions of burnout are comparable across national contexts. In sum, although men and women, as well as employees from different countries, may differ in their average levels of burnout, the mechanisms underlying burnout are likely to operate similarly across groups. Therefore, we hypothesize that

**Hypothesis** **3** **(H3).** *The proposed burnout–emotion model will be invariant across gender.*

**Hypothesis** **4** **(H4).** *The proposed burnout–emotion model will be invariant across countries.*

### 1.2. Guilt as a Moderator in the Burnout-Emotion Process

Guilt is a self-referential moral emotion that arises from the perception of having transgressed personal or social norms, with ambivalent implications for psychological well-being (Baumeister et al., 1994). In the occupational context, guilt has been recognized as a secondary symptom emerging in the later stages of the burnout process (Gil-Monte, 2019). For instance, Gil-Monte (2005) proposed the existence of two distinct burnout profiles: Profile 1, characterized by low levels of guilt and a functional coping strategy based on emotional distancing; and Profile 2, characterized by high levels of guilt, which may contribute to deteriorating health and an increased desire to leave the profession (Guidetti et al., 2018). In addition, guilt has been conceptualized as a mediating variable that emerges as an emotional response to dysfunctional coping strategies. Its inclusion has contributed to a more nuanced understanding of the burnout process by linking antecedents to outcomes (Figueiredo-Ferraz et al., 2021; Gil-LaOrden et al., 2024).

However, its previously established mediating role does not preclude guilt from also operating as a moderator of other relationships within the burnout process. Accordingly, the present study addresses a different question: whether guilt changes the strength of the association between the core burnout syndrome and emotional experiences at work. According to appraisal theory (Lazarus, 1982), guilt signals that one’s behavior has violated a personal standard, triggering a secondary appraisal of available coping resources. When burnout is high, individuals with low levels of guilt may appraise their emotional exhaustion as externally caused, allowing them to disengage emotionally and thereby amplifying the reduction in positive emotions. In contrast, individuals with high levels of guilt remain self-focused and engaged in reparative cognition (Tangney et al., 2007), which may paradoxically attenuate the linear decline in positive emotions even as burnout escalates.

Put differently, guilt may function as a cognitive brake that prevents complete affective disengagement: the individual still experiences distress, but the pattern of emotional deterioration is altered. A parallel mechanism may operate for negative emotions. Individuals with high levels of guilt may already carry a chronic emotional burden at baseline, leaving less room for burnout to generate additional distress, a pattern consistent with a possible ceiling effect (Gross & John, 2003). Building on this reasoning, we propose that guilt operates not only as an outcome of burnout but also as a moderator of its emotional consequences.

**Hypothesis** **5** **(H5).** *Guilt will moderate the relationship between burnout and positive emotions, such that the negative association will be stronger when guilt is low and weaker when guilt is high.*

**Hypothesis** **6** **(H6).** *Guilt will moderate the relationship between burnout and negative emotions, such that the positive association will be stronger when guilt is low and weaker when guilt is high.*

## 2. Materials and Methods

### 2.1. Sample and Procedure

This study was approved by the Research Ethics Committee of the Valencian International University (protocol code CEID2021_14), ensuring compliance with ethical standards and research integrity. Following ethical approval, data were collected using a snowball sampling technique. An online questionnaire was distributed through organizational intranets and social media platforms between April and September 2022. A total of 857 valid questionnaires were obtained from employees across four Ibero-American countries (Spain, *n* = 196; Chile, *n* = 232; Colombia, *n* = 222; and Mexico, *n* = 207).

Regarding the characteristics of the sample, 57.2% of participants were women. Participants ranged in age from 18 to 65 years (M = 38.50, SD = 10.55). Although the average organizational tenure was 72.5 months (SD = 85.4), the most frequently represented category was 0–12 months (28.8%). In terms of educational attainment, most participants held higher education qualifications, with 39.3% reporting a bachelor’s degree, 29.9% a master’s degree, and 4.4% a doctoral degree. Additionally, 16.9% reported vocational or technical education, whereas lower levels of formal education were minimally represented in the sample. The sample was multi-occupational; however, detailed occupational categories were not collected, and therefore participants could not be characterized according to occupational group.

To encourage participation, participants were entered into a lottery offering a small financial incentive (€20). The draw was conducted after the completion of data collection.

### 2.2. Instruments

Emotions at work were measured using six workplace emotions (one item per emotion): three positive emotions (i.e., cheerful, optimistic, enthusiastic; α = 0.92) and three negative emotions (i.e., tense, worried, uneasy; α = 0.88; Segura & González-Romá, 2003). Participants were asked to rate each emotion on a 5-point scale ranging from 0 (Never) to 4 (Always), according to how their work made them feel.

Burnout was assessed using the Spanish Burnout Inventory (Gil-Monte, 2019). The burnout score comprises 15 items distributed across three core subscales: (1) Enthusiasm toward the Job (5 items; e.g., “I see my job as a source of personal accomplishment”; α = 0.90); (2) Psychological Exhaustion (4 items; e.g., “I feel emotionally exhausted”; α = 0.85); and (3) Indolence (6 items; e.g., “I think I treat some of the people I interact with at work with indifference”; α = 0.83). Responses were provided on a 5-point Likert-type scale ranging from 0 (Never) to 4 (Every day). According to the SBI theoretical and scoring model (Gil-Monte, 2011), the overall burnout score is derived from these three core dimensions—low enthusiasm toward the job, psychological exhaustion, and indolence.

Guilt was assessed using the Guilt subscale of the Spanish Burnout Inventory (Gil-Monte, 2019), which comprises 5 items (e.g., “I regret some of my behaviors at work”; α = 0.82). Responses were provided on the same 5-point Likert-type scale ranging from 0 (Never) to 4 (Every day). Importantly, guilt is not included in the overall burnout score; it is assessed separately and is used to distinguish between the two burnout profiles (Gil-Monte, 2011).

### 2.3. Data Analysis

First, sample size adequacy, normality assumptions, reliability, descriptive statistics, and correlation analyses were examined prior to conducting the main analyses. As a diagnostic check for potential common method bias, a single-factor CFA was estimated in which the nine observed indicators included in the structural model were constrained to load onto a single latent factor.

In accordance with the SBI scoring model, burnout and guilt were treated as separate variables throughout the analyses. In the SEM analyses, burnout was specified as a latent construct indicated by Enthusiasm toward the Job, Psychological Exhaustion, and Indolence. Because higher Enthusiasm toward the Job reflects lower burnout, this dimension loaded negatively on the latent burnout factor. Guilt was not included as an indicator of the burnout factor. In the PROCESS moderation analyses, the observed overall burnout score was computed according to the SBI scoring procedure. Specifically, the five Enthusiasm toward the Job items were reverse scored and combined with the four Psychological Exhaustion items and the six Indolence items. The 15 items were then averaged to obtain the overall burnout score. The five Guilt items were not included in this score, and guilt was entered separately as the moderator. Therefore, the burnout × guilt interaction involved two mathematically non-overlapping variables.

Second, to test the hypotheses, structural equation modelling (SEM) was conducted using AMOS (Arbuckle, 2010) to examine the relationships between burnout and emotional outcomes (i.e., cheerful, optimistic, enthusiastic, tense, worried, and uneasy). To evaluate model fit, several goodness-of-fit indices were examined: (1) the χ^2^ goodness-of-fit statistic; (2) the root mean square error of approximation (RMSEA); (3) the Normed Fit Index (NFI); (4) the Tucker–Lewis Index (TLI); (5) the Incremental Fit Index (IFI); (6) the Comparative Fit Index (CFI); and (7) Akaike’s Information Criterion (AIC). Model fit was evaluated using multiple complementary indices. Given the well-known sensitivity of the χ^2^ statistic to sample size, model adequacy was not determined on the basis of χ^2^ significance alone. RMSEA values below 0.06 were interpreted as indicating good fit. For incremental fit indices, values above 0.95 were interpreted as indicating excellent fit, whereas values above 0.90 were considered acceptable (Hu & Bentler, 1999).

Third, a multigroup SEM analysis was conducted to examine the robustness of the proposed structural model across gender and countries. Following Cheung and Rensvold (2002), configural (i.e., same factor structure across groups), metric (i.e., same factor loadings across groups), scalar (i.e., same item intercepts across groups), and strict (i.e., same residual variances across groups) invariance models were assessed by comparing each model’s fit with that of the preceding model. Invariance at each step was considered to be supported when changes in model fit remained within the recommended cut-off values: Δχ^2^ (*p* > 0.05), ΔCFI ≤ 0.01, and ΔRMSEA ≤ 0.015. However, CFI was considered the primary criterion for evaluating invariance (Milfont & Fischer, 2010; Putnick & Bornstein, 2016), given that the χ^2^ statistic is highly sensitive to large sample sizes and may therefore be less reliable when samples exceed 200 participants (Cheung & Rensvold, 2002; Hu & Bentler, 1999).

Finally, moderation analyses were conducted using PROCESS Macro version 4.2 (Hayes, 2022) to examine whether guilt altered the relationship between burnout and emotions at work. In these models, burnout was specified as the independent variable, positive and negative emotions as the dependent variables, and guilt as the moderator. Conditional effects were examined at low (16th percentile), median (50th percentile), and high (84th percentile) levels of guilt.

## 3. Results

### 3.1. Preliminary Analyses

Based on recommendations for sample size estimation in structural equation modelling (SEM; Soper, 2023), a minimum sample size of 200 participants was required to test the proposed model, which included 9 observed variables and 3 latent variables, assuming an anticipated effect size of 0.30, a significance level of 0.05, and a statistical power of 0.80. The present study included 857 participants, substantially exceeding the minimum sample size requirement for SEM analyses.

First, Table 1 presents the means, standard deviations, skewness, kurtosis, and Pearson correlations among the study variables. All variables were significantly correlated in the expected directions. In addition, Cronbach’s alpha coefficients indicated adequate internal consistency for burnout, positive emotions, negative emotions, and guilt. In addition, the single-factor CFA showed a poor fit to the data, χ^2^(27) = 1374.33, *p* < 0.001, CFI = 0.718, TLI = 0.625, and RMSEA = 0.241, indicating that a single common factor did not adequately account for the covariance among the observed indicators.

### 3.2. Hypothesis Testing: SEM

**Figure 1:**
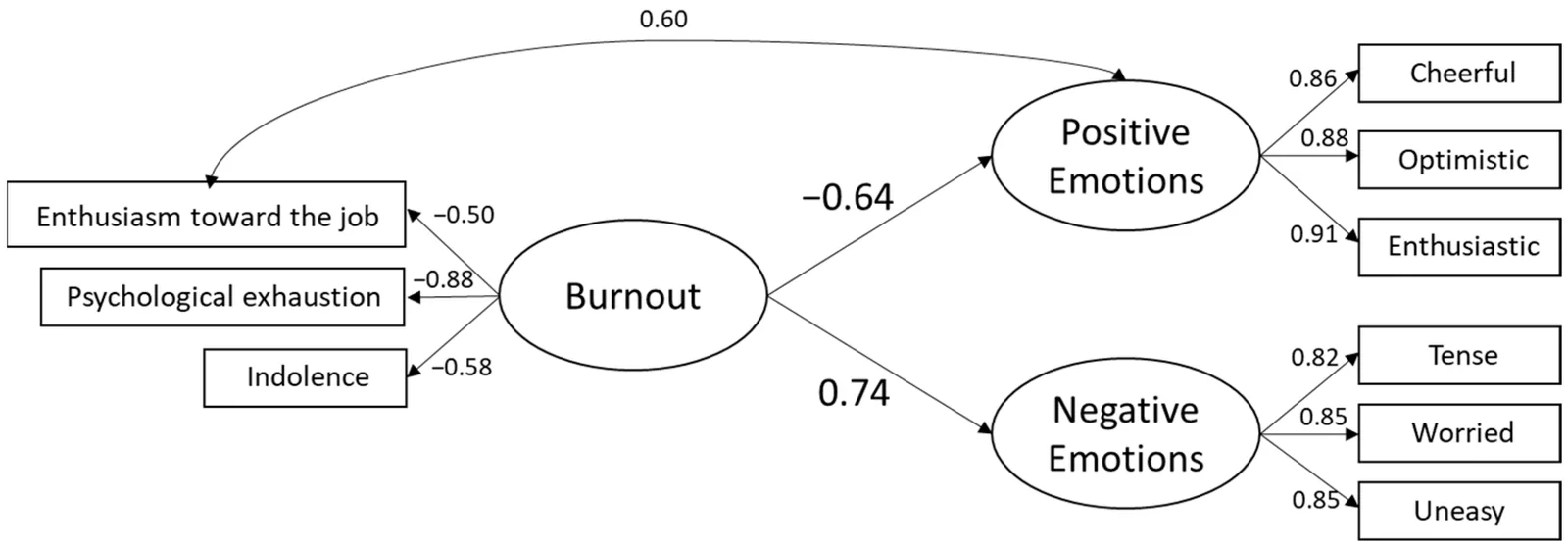
Presents the standardized path coefficients.

The initial model, estimated without allowing correlated residuals, showed inadequate fit on several indices: [χ^2^(25) = 289.54, *p* < 0.001, RMSEA = 0.111, CFI = 0.945, NFI = 0.940, TLI = 0.920, IFI = 0.945, AIC = 347.542]. Examination of the modification indices revealed a substantial residual covariance between the measurement error associated with Enthusiasm toward the Job and the disturbance term of the Positive Emotions latent factor (M.I. = 155.810; parameter change = 0.194). Although Enthusiasm toward the Job is an indicator of burnout, it reflects a positive affective–motivational evaluation of the job (e.g., feeling stimulated, gratified, and perceiving the job as a source of positive outcomes), which is conceptually related to positive emotional experiences such as feeling cheerful, optimistic, and enthusiastic. However, the two constructs remain theoretically distinct: Enthusiasm toward the Job is anchored in work motivation and job appraisal, whereas Positive Emotions reflect momentary affective states that are not necessarily tied to evaluations of one’s job. Accordingly, a theoretically justified residual covariance was specified to account for shared affective variance not fully captured by the burnout factor, without collapsing the two constructs into a single factor. This specification reflects the presence of unmodeled affective processes common to both constructs and does not imply a direct causal relationship between them. Furthermore, allowing this residual covariance resulted in a substantial improvement in model fit and is consistent with SEM recommendations when localized misfit reflects theoretically interpretable shared variance (Byrne, 2010; Kline, 2016; MacCallum, 1986). The re-specified model demonstrated a substantially better fit to the data: [χ^2^(24) = 47.06, *p* = 0.003, RMSEA = 0.034, CFI = 0.995, NFI = 0.990, TLI = 0.993, IFI = 0.995, AIC = 107.056]. See Table 2. The standardized path coefficients were also compared across the initial and re-specified models. The paths from burnout to positive and negative emotions changed from β = −0.80 and β = 0.70, respectively, to β = −0.64 and β = 0.74, indicating that the re-specification did not systematically inflate the structural estimates. See Figure 1.

### 3.3. Hypothesis Testing: Multigroup SEM

To examine whether the proposed model operated similarly across gender and countries, two multigroup SEM analyses were conducted, using gender and country as grouping variables, respectively.

With regard to gender, the results supported configural, metric, scalar, and strict invariance. Changes in model fit remained below the recommended cut-off values (ΔCFI ≤ 0.01; ΔRMSEA ≤ 0.015), indicating that the measurement model operated equivalently across men and women. Although the χ^2^ difference test for Model 3 (scalar invariance) was statistically significant, the model was retained because χ^2^ is known to be highly sensitive to large sample sizes (Cheung & Rensvold, 2002; Hu & Bentler, 1999). Moreover, both ΔCFI and ΔRMSEA remained within the recommended thresholds, supporting the conclusion that scalar invariance was achieved. See Table 3.

With respect to country, the results supported configural and metric invariance, indicating that the factor structure and factor loadings of burnout, positive emotions, and negative emotions were comparable across countries. However, scalar and strict invariance were not supported. These findings suggest that although the constructs were conceptualized similarly across national contexts and the relationships among indicators and latent variables were equivalent, differences in item intercepts and residual variances remained across countries. See Table 4.

### 3.4. Hypothesis Testing: Moderation Analysis

Finally, to determine whether the association between burnout and emotions varied as a function of guilt, moderation analyses were conducted using linear regression. Specifically, we examined whether guilt altered the strength of the relationship between burnout and emotional outcomes.

Results revealed that the interaction between burnout and guilt was a significant predictor of positive emotions (B = 0.229, SE = 0.059, t = 3.87, *p* < 0.001, 95% CI [0.113, 0.345]), explaining an additional 1.67% of variance beyond the main effects (ΔR^2^ = 0.017). Likewise, the interaction significantly predicted negative emotions (B = −0.194, SE = 0.066, t = −2.93, *p* = 0.003, 95% CI [−0.324, −0.064]), explaining an additional 0.81% of variance (ΔR^2^ = 0.008).

**Figure 2:**
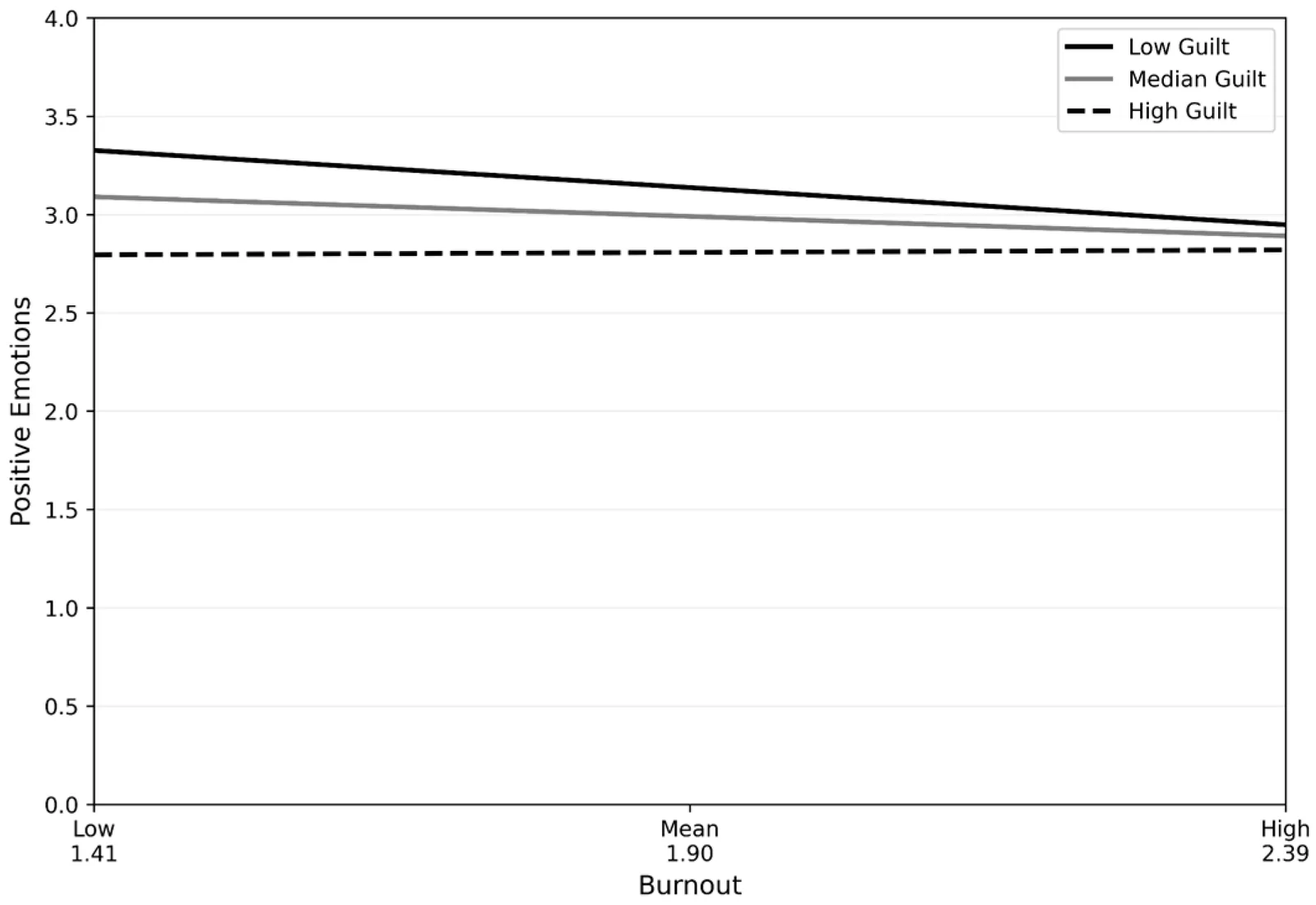
Simple-slope analysis of the interaction between burnout and guilt predicting positive emotions. Values on the Y-axis represent predicted mean positive-emotion scores on the original 0–4 response scale. Low, median, and high guilt correspond to the 16th, 50th, and 84th percentiles of the observed guilt distribution, respectively. Burnout values displayed correspond to M − 1 SD (1.41), M (1.90), and M + 1 SD (2.39).

As illustrated in Figure 2, burnout was negatively associated with positive emotions, and this relationship was stronger when guilt was low, whereas it was attenuated at higher levels of guilt. Although individuals with high levels of guilt reported lower levels of positive emotions overall, the decline in positive emotions associated with increasing burnout was less pronounced in this group. Indeed, at high levels of guilt, the relationship between burnout and positive emotions was no longer statistically significant. Specifically, the conditional effect decreased from b = −0.386 (*p* < 0.001) at low guilt to b = −0.203 (*p* = 0.004) at median guilt and b = 0.026 (*p* = 0.759) at high guilt.

**Figure 3:**
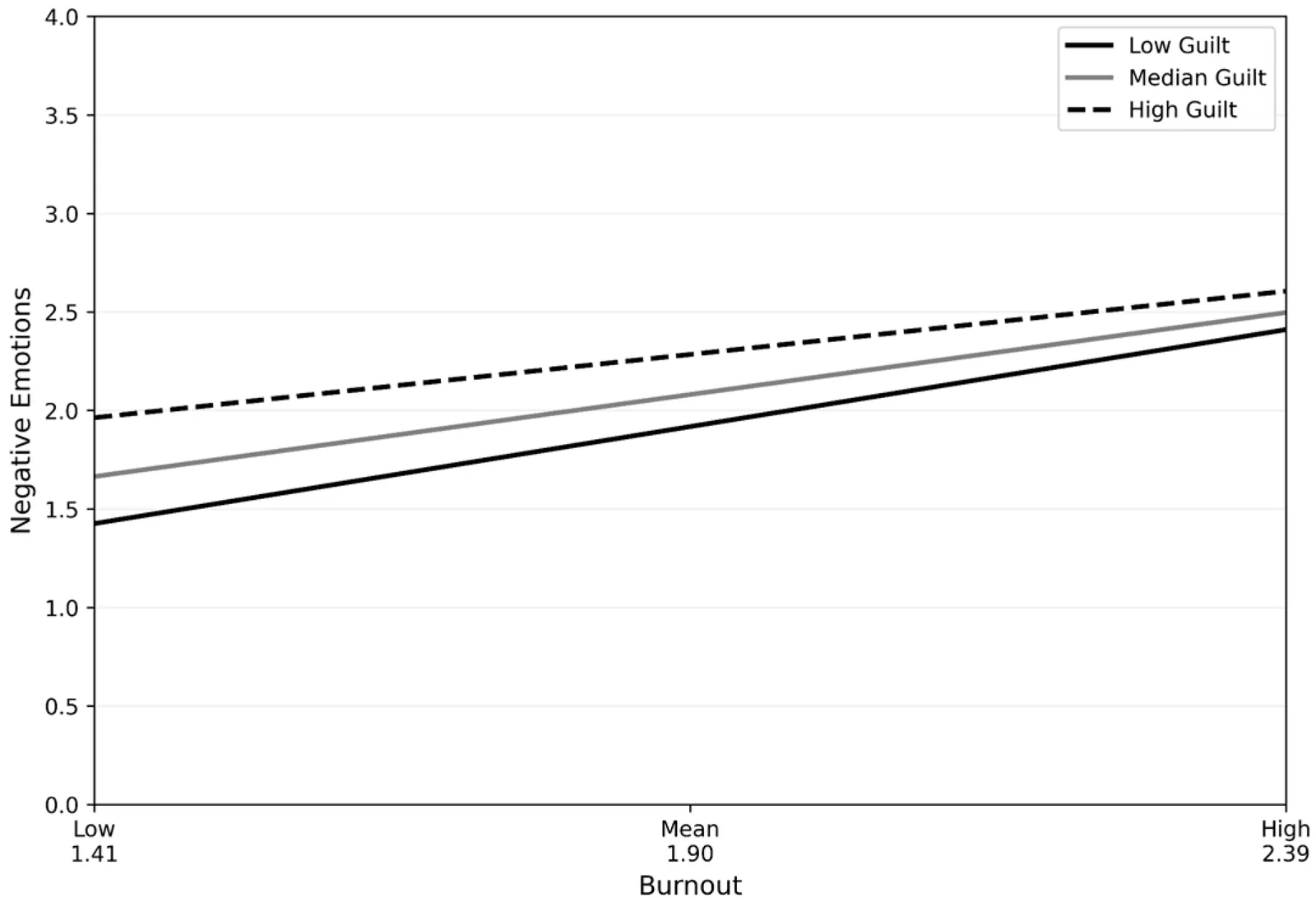
Simple-slope analysis of the effect of the interaction between burnout and guilt on negative emotions. Values on the Y-axis represent predicted mean negative-emotion scores on the original 0–4 response scale. Low, median, and high guilt correspond to the 16th, 50th, and 84th percentiles of the observed guilt distribution, respectively. Burnout values displayed correspond to M − 1 SD (1.41), M (1.90), and M + 1 SD (2.39).

Similarly, burnout was positively associated with negative emotions, with steeper increases observed among individuals with low levels of guilt (see Figure 3). By contrast, when guilt was high, negative emotions were already elevated at low levels of burnout, and the incremental effect of burnout on negative emotions was weaker. The conditional effect decreased from b = 1.005 (*p* < 0.001) at low guilt to b = 0.850 (*p* < 0.001) at median guilt and b = 0.655 (*p* < 0.001) at high guilt. Taken together, these findings indicate that guilt moderates the relationship between burnout and emotional experiences by influencing the strength, rather than the direction, of these associations.

## 4. Discussion

The aim of the present study was to examine the relationship between burnout and emotions at work, as well as the moderating role of guilt in this process, using a cross-national sample of employees from four Ibero-American countries (Spain, Chile, Colombia, and Mexico).

First, consistent with Gil-Monte’s (2005) theoretical model of burnout, the results provided full support for Hypotheses 1 and 2. Employees reporting higher levels of burnout experienced fewer positive emotions and more negative emotions at work. These findings are consistent with previous research documenting close associations between burnout and emotional experiences in occupational settings (Atmaca et al., 2020; Zapf et al., 2001), and they support the direction of the burnout–emotion relationship proposed by Burić et al. (2019). The standardized path coefficients obtained (β = −0.64 for positive emotions and β = 0.74 for negative emotions) indicate not only statistically significant but also substantial associations, underscoring the practical relevance of the relationship between burnout and employees’ emotional experiences at work.

Second, the multigroup analyses provided evidence regarding the generalizability of the proposed model. On the one hand, strict invariance across gender suggests that the relationships among burnout, positive emotions, and negative emotions operate similarly for men and women. This finding is consistent with previous studies indicating that although mean levels of burnout may differ slightly across genders, the underlying psychological processes are largely comparable (Osca et al., 2024; Purvanova & Muros, 2010; Umaña-Ruiz et al., 2023). On the other hand, configural and metric invariance across countries indicate that the factor structure and the strength of the relationships among constructs are equivalent across Spain, Chile, Colombia, and Mexico. However, the absence of scalar and strict invariance suggests that participants from different countries may differ in baseline levels of the variables or in the amount of unexplained variance associated with them. In sum, our findings provided full support for Hypothesis 3 and partial support for Hypothesis 4.

Finally, our findings provided full support for Hypotheses 5 and 6, revealing that guilt moderated the relationship between burnout and emotions at work. Although guilt has traditionally been conceptualized as a symptom of burnout and a marker of more severe burnout profiles (Gil-Monte, 2005, 2019; Guidetti et al., 2018), the present findings suggest a more complex role for guilt in the burnout process. Specifically, guilt may function not only as an outcome of burnout (Figueiredo-Ferraz et al., 2021; Gil-LaOrden et al., 2024), but also as a variable that shapes its emotional consequences (Gross & John, 2003; Tangney et al., 2007).

Although guilt has been associated with rumination and heightened distress in other contexts (Gross & John, 2003), the present findings consistently revealed an attenuation pattern: higher levels of guilt were associated with weaker relationships between burnout and both negative and positive emotions across increasing levels of burnout. This suggests that, in the occupational context, the self-regulatory and reparative functions of guilt (Tangney et al., 2007) may predominate over its ruminative aspects.

One possible interpretation is that, at low levels of guilt, the absence of self-evaluative pressure may facilitate emotional disengagement and the externalization of responsibility, mechanisms consistent with Profile 1 described by Gil-Monte (2005). This interpretation is consistent with the possibility that affective detachment may be more strongly associated with emotional deterioration when guilt is low. Conversely, at high levels of guilt, the self-regulatory and reparative cognitions associated with this emotion (Tangney et al., 2007) may be linked to a weaker association between burnout and emotional deterioration. Although these mechanisms were not directly assessed in the present study, they offer a plausible theoretical interpretation of the observed moderation effects.

Importantly, this attenuation pattern does not imply that high levels of guilt are without costs. Gil-Monte’s Profile 2 describes a pattern in which guilt is associated with more severe burnout profiles, including psychosomatic symptoms and turnover intentions (Guidetti et al., 2018; Gil-LaOrden et al., 2024). The present findings suggest that guilt may operate differently depending on the outcome considered. In relation to emotional experiences, guilt was associated with weaker relationships between burnout and both positive and negative emotions. However, previous research has linked guilt to more severe burnout profiles and psychosomatic outcomes. Taken together, these findings support the view that guilt may play a complex role within the burnout process, acting as a boundary condition of burnout-related emotional experiences while also being associated with indicators of more severe burnout. As a chronic feature of the burnout process, however, it may contribute to more severe long-term consequences. Therefore, guilt may be associated with a weaker relationship between burnout and emotional experiences while simultaneously being linked, according to previous research, to indicators of more severe burnout profiles.

### 4.1. Theoretical and Practical Implications

This study makes several contributions to the burnout literature. First, it advances current knowledge regarding the relationship between burnout and emotions by supporting a less frequently examined perspective. Whereas previous studies have predominantly focused on emotions as antecedents of burnout (Atmaca et al., 2020; Szczygieł & Mikołajczak, 2018; Zapf et al., 2001), the present findings support the theoretical proposition that burnout is closely associated with employees’ emotional experiences and are consistent with the possibility that burnout may contribute to these emotional outcomes.

Second, Gil-Monte and colleagues (Gil-Monte, 2019; Guidetti et al., 2018; Figueiredo-Ferraz et al., 2021; Gil-LaOrden et al., 2024) proposed that guilt functions as an emotional response to dysfunctional coping strategies, particularly indolence. The present study extends this proposition by positioning guilt as a boundary condition of the emotional consequences of burnout. Specifically, the findings demonstrate that the intensity, rather than the direction, of burnout-related emotional deterioration varies according to employees’ levels of guilt.

From a practical perspective, the findings suggest several promising directions for burnout prevention and intervention. On the one hand, organizations should pay greater attention to employees’ emotional experiences by promoting healthy and supportive leadership practices. For example, transformational leaders foster positive work environments by providing support, communicating an inspiring vision, and addressing employees’ individual needs, all of which have been associated with higher levels of well-being and lower levels of burnout (Pladdys, 2024).

On the other hand, both psychotherapy and Employee Assistance Programs (Solé & Balduque, 2006) may benefit from considering factors such as guilt and employees’ cultural context, as these variables may influence how burnout develops and how its consequences are experienced and prevented.

### 4.2. Limitations and Future Studies

Some limitations of the present study should be noted. First, a non-probabilistic convenience sample was used, which may limit the generalizability of the findings to broader working populations. Future studies should employ probability-based sampling strategies to enhance external validity.

Second, detailed occupational information was not collected. Consequently, the sample could not be characterized by occupational group, and occupation could not be included as a control variable in the analyses. Given that burnout levels may vary across professions and sectors (García-Carmona et al., 2019; Giménez-Bertomeu et al., 2024; Woo et al., 2020), occupational heterogeneity may have introduced additional unexplained variance into the observed relationships. Future studies should collect detailed occupational information and examine whether the proposed associations vary across occupational groups or specific professional contexts.

Third, data were collected exclusively through self-report measures, which may introduce common method bias. Although this approach is appropriate for assessing subjective psychological states (Corneille & Gawronski, 2024), future studies could complement self-reports with physiological indicators of emotional arousal or peer-rated assessments of emotional expression. Fourth, the cross-sectional design does not allow firm conclusions about the causal ordering among the variables studied. This is particularly relevant because burnout and emotions may be part of reciprocal gain and loss spirals, in which emotional experiences function both as antecedents and consequences of burnout (Llorens & Salanova, 2014). Longitudinal and experience-sampling designs would allow these dynamic relationships to be examined with greater precision. Fifth, scalar and strict invariance across countries was not fully supported, suggesting that participants from different national contexts may differ in baseline levels or residual variance. This limits the comparability of latent mean differences across countries and should be addressed in future cross-cultural research with larger and more balanced national samples.

Finally, future studies should examine how burnout-related emotional experiences interact with other relevant variables, such as job crafting (Pijpker et al., 2022) or self-care behaviours (González-Moreno et al., 2025), which may further clarify the circumstances under which burnout is associated with employees’ emotional well-being.

## Figures and Tables

**Table 1:** Descriptive and bivariate correlations between variables (*N* = 857).

Variables	M	SD	S	K	1	2	3	4	5	6	7	8
1. Cheerful	3.3	0.95	−0.83	0.23	-	0.76 **	0.79 **	−0.37 **	−0.33 **	−0.34 **	−0.14 **	−0.12 **
2. Optimistic	3.2	1.0	−0.95	0.4	-	-	0.88 **	−0.39 **	−0.35 **	−0.37 **	−0.14 **	−0.14 **
3. Enthusiastic	2.99	0.97	−0.84	0.27	-	-	-	−0.41 **	−0.38 **	−0.39 **	−0.15 **	−0.13 **
4. Tense	2.27	1.14	−0.8	−0.79	-	-	-	-	0.70 **	0.69 **	0.24 **	0.38 **
5. Worried	1.87	1.21	0.19	−0.85	-	-	-	-	-	0.74 **	0.29 **	0.36 **
6. Uneasy	2.7	1.32	0	−1.13	-	-	-	-	-	-	0.29 **	0.38 **
7. Guilt	0.92	0.83	0.94	0.47	-	-	-	-	-	-	-	0.49 **
8. Burnout	1.9	0.49	0.52	0.8	-	-	-	-	-	-	-	-

Note: M = Mean, SD = Standard Deviation, S = Skewness; K = Kurtosis; ** *p* < 0.01.

**Table 2:** Comparison of model fit and standardized structural path coefficients for the initial and re-specified SEM models.

Model	χ^2^	df	RMSEA	CFI	NFI	TLI	IFI	AIC	β Burnout → P. Emotions	β Burnout → N. Emotions
Initial model	289.54	25	0.111	0.945	0.940	0.920	0.945	347.542	−0.80	0.70
Re-specified model	47.06	24	0.034	0.995	0.990	0.993	0.995	107.056	−0.64	0.74

**Table 3:** Tests of Measurement Invariance across Gender.

Invariance Test	X^2^	df	X^2^/df	CFI	RMSEA	Model Comparison	ΔX^2^	ΔCFI	ΔRMSEA	Decision
M1. Configural (Unconstrained)	67.507	48	1.406	0.996	0.022	–	–	–	–	Accept
M2. Metric (Measurement weights)	71.677	54	1.327	0.996	0.020	M1	4.170 (6), *p* = 0.653	0.005	0.002	Accept
M3. Scalar (Measurement intercepts)	115.238	63	1.929	0.989	0.031	M2	43.561 (9), *p* < 0.001	0.007	0.011	Accept
M4. Strict (Measurement residuals)	133.717	78	1.714	0.988	0.029	M3	18.479 (15), *p* = 0.238	0.001	0.002	Accept

**Table 4:** Tests of Measurement Invariance across Countries.

Invariance Test	X^2^	df	X^2^/df	CFI	RMSEA	Model Comparison	ΔX^2^	ΔCFI	ΔRMSEA	Decision
M1. Configural (Unconstrained)	171.202	96	1.783	0.985	0.030	–	–	–	–	Accept
M2. Metric (Measurement weights)	209.581	114	1.838	0.980	0.031	M1	38.379 (18), *p* = 0.003	0.005	0.001	Accept
M3. Scalar (Measurement intercepts)	342.327	141	2.428	0.959	0.041	M2	132.746 (27), *p* < 0.001	0.021	0.010	Reject
M4. Strict (Measurement residuals)	536.942	186	2.887	0.928	0.047	M3	194.615 (45), *p* < 0.001	0.031	0.006	Reject

## Data Availability

The raw data supporting the conclusions of this article will be made available by the authors upon request.
